# Analysing biological colour patterns from digital images: An introduction to the current toolbox

**DOI:** 10.1002/ece3.11045

**Published:** 2024-03-18

**Authors:** Christopher R. Hemingson, Peter F. Cowman, David R. Bellwood

**Affiliations:** ^1^ The Research Hub for Coral Reef Ecosystem Functions James Cook University Townsville Queensland Australia; ^2^ College of Science and Engineering James Cook University Townsville Queensland Australia; ^3^ Biodiversity and Geosciences Program, Queensland Museum Tropics Townsville Queensland Australia

**Keywords:** approaches, colouration, colours, guide, image analysis, methods, patterns, tools

## Abstract

Understanding the numerous roles that colouration serves in the natural world has remained a central focus in many evolutionary and ecological studies. However, to accurately characterise and then compare colours or patterns among individuals or species has been historically challenging. In recent years, there have been a myriad of new resources developed that allow researchers to characterise biological colours and patterns, specifically from digital imagery. However, each resource has its own strengths and weaknesses, answers a specific question and requires a detailed understanding of how it functions to be used properly. These nuances can make navigating this emerging field rather difficult. Herein, we evaluate several new techniques for analysing biological colouration, with a specific focus on digital images. First, we introduce fundamental background knowledge about light and perception to be considered when designing and implementing a study of colouration. We then show how numerous modifications can be made to images to ensure consistent formatting prior to analysis. After, we describe many of the new image analysis approaches and their respective functions, highlighting the type of research questions that they can address. We demonstrate how these various techniques can be brought together to examine novel research questions and test specific hypotheses. Finally, we outline potential future directions in colour pattern studies. Our goal is to provide a starting point and pathway for researchers wanting to study biological colour patterns from digital imagery.

## Graphical abstract

We provide an introduction to the study of biological colour patterns using digital imagery. We cover many of the new techniques and describe their strength and weakness. This resource acts as a springboard for researchers entering the field.

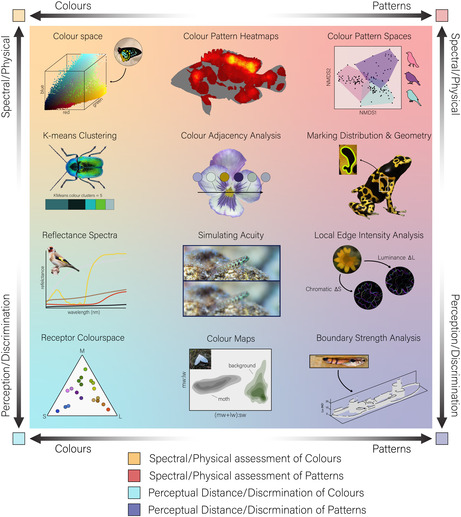

## 1 INTRODUCTION

Understanding the role that certain colours and patterns serve in biological systems has remained a central focus in evolutionary and ecological studies. An organism's colouration (the combination of colours and patterns) often has an intrinsic link to its life history strategy; dictating how it behaves and interacts with other organisms as well as its environment. Researchers and naturalists alike have been fascinated with the intricacies of animal colouration since the times of Darwin and Wallace (Caro, 2017; Darwin, 1859; Wallace, 1877). However, the physical properties that are responsible for creating colouration makes it difficult to objectively study (Endler, 1978). How light behaves and interacts within an environment is extremely context dependent. Furthermore, how this light is then subsequently perceived and processed by another organism makes this seemingly simple field rather complex (Endler, 1990).

Darwin and Wallace would likely be impressed with the progress that has been made in characterising and quantifying organismal colours and patterns (Endler, 1978, 1990). Historically, descriptions of colouration were both context and viewer dependent. As noted by Longley, 1917: ‘The method is crude; allowance for the personal equation of the observer must be large…’. The advent of spectrometers, which operate by detecting the intensity of light at different wavelengths, allowed for more physical descriptions of light and consequently colour, to be made (Endler, 1990; Johnsen, 2016). Reflectance spectra can tell us detailed information about the object being measured, for example, which pigments are likely responsible for creating a specific colour (Toral et al., 2008). While this is by far the most accurate method for assessing the colour of an object, it does have its disadvantages. Reflectance spectra must be remeasured for each specific colour of interest making data collection both labour and equipment intensive (Marshall et al., 2003). In the life sciences, this means the observer must also decide which parts of the organism's body and pattern to measure, imposing a bias as to which aspects of colouration are thought to be meaningful (Badiane et al., 2017; Dalrymple et al., 2015). Importantly, they fail to provide any description of patterns, leaving this completely up to the interpretation of the viewer.

However, digital images provide an ideal medium in which to study biological colour patterns (Stevens et al., 2007). Since images inherently record spatial information of colour (i.e. its pattern), they are well suited for characterising the colour pattern data. Digital images remove the subjectivity of classifying patterns based on human constructs (e.g. categorising a pattern as ‘stripes’ or ‘spots’) and do not require the user to specify locations on an organism that has been deemed important for measurement. Furthermore, the relatively cheaper cost of many digital cameras compared to a complete spectrometer setup and their ease of use in the field make them a valuable resource for colour pattern studies.

In recent years, there has been a surge of new methodologies that aim to describe and characterise biological colour patterns, specifically from digital imagery (Mason & Bowie, 2020). These methods have benefitted from the combination of more informed research designs and affordable computing. Through the advent of open source programming languages, like R (R Core Team, 2023), many new and free computational resources are now available for use. These new resources allow researchers to ask and answer questions that were previously not possible. However, each technique or application possesses its own strengths and weaknesses, answers specific questions and requires time to learn and implement.

Herein, we present an introduction to many of the recent tools available for analysing biological colour patterns and their application. The resources covered will primarily focus on image analysis techniques that are available in open‐source, user‐friendly software, as these are the methods that have seen the most recent growth. First, we detail the basic knowledge around colouration and vision and highlight some key considerations to be made when constructing a study. We then provide an overview of what resources are available to measure and characterise colours and patterns from digital images. Finally, we demonstrate how some of these various techniques can be brought together and describe their potential applicability by outlining future directions for colour research. Our overall aim is to provide a resource for researchers entering the field of colour pattern science to help design, develop and conduct studies on biological colouration using new techniques in a rapidly growing field.

## 2 METHODOLOGICAL APPROACHES

### 2.1 Vision and perception: a necessary primer

Colours and patterns are a product of light and its ability to be detected, processed and interpreted by a viewer. Therefore, a fundamental understanding of both the physical properties of light and how it is viewed and processed is essential to study biological colourations. Light is electromagnetic radiation (small quantities of energy that lack mass or charge) that behaves in some manner as both a particle and wave. Visible light refers to the spectrum of electromagnetic radiation visible to most humans which spans from approximately 380 to 750 nm in wavelength (wavelength is frequently denoted by the symbol *λ*). However, many organisms can detect light in the ultraviolet range (300–400 nm; Siebeck, 2004) or shortwave infrared (750 nm–1000 nm; Gracheva et al., 2010), which is important to consider if your study explicitly involves a known, non‐human viewer (Caves et al., 2019). Light is detected in the retina of the eye by two main photoreceptor cell types: rods and cones. Rods are primarily involved in detecting changes in luminance, i.e. light intensity. Thus, rods are generally used for low light or night vision and do not often decipher chromatic (colour) differences. Conversely, cone cells are involved in the detection of light with difference wavelengths (i.e. colours) and have a greater variety of cell types which are often tuned to different spectral sensitivities. These different spectral sensitivities are determined by the type of opsin protein expressed by the photoreceptor; opsins being the light sensitive protein that react to light stimulus ultimately starting the colour detection and processing pathway for many organisms (Shichida & Matsuyama, 2009). Light of different wavelengths appear different in colour depending on the filtering media within the lens (e.g. oil droplets; Vorobyev, 2003), the type, density and the orientation of photoreceptors in the retina of the viewer (Carleton et al., 2020), as well as how an organism neurally processes the light signal (Endler, 1990). For comprehensive reviews on the acquisition and neural processing of light, see (Endler, 1978, 1990; Kelber et al., 2003; Kemp et al., 2015; Osorio & Vorobyev, 2008).

How do we make accurate assumptions about what colours other organisms can perceive? These conclusions are made through either: (1) behavioural experiments, (2) measurements of certain cellular properties within their retina (microspectrophotmetry and electroretinography) or, (3) more recently, by identifying genetic sequences that are commonly known to code for visual opsin proteins – the light sensitive proteins that are universal in animal vision (Kelber et al., 2003; Kemp et al., 2015; Shichida & Matsuyama, 2009). Behavioural experiments typically present a study organism with different stimuli to observe perceptive abilities and test their responses (Newport et al., 2017; Siebeck et al., 2008). Microspectrophotmetry and electroretinography work by either measuring the amount of light absorbed by photoreceptor cells or by measuring the electrical activity within the retina. Both techniques provide evidence as to what wavelengths of light the organisms likely can or cannot see, however exceptions do occur (Losey et al., 2003; Tosetto et al., 2021). Last, dedicated genetic research has linked certain gene encoding regions to the expression of specific visual opsin proteins. Opsins are a class of light sensitive proteins which give certain photoreceptor cells their ability to detect light. Different opsins have different spectral sensitivities they react to. Therefore, by identifying which opsins are being coded for, we can infer what possible spectral sensitivities an organism may have (Carleton et al., 2020; Musilova et al., 2019). It is important to note that the presence of specific opsin encoding genes does not directly equate to an individual possessing photoreceptors with that protein as organism's may ‘tune’ their visual capabilities to best fit the corresponding light environment the organism resides within (Kranz et al., 2018; Nandamuri et al., 2017). Ultimately, each of these approaches provides evidence as to what an organism likely can or cannot perceive. The latter of these two techniques must be validated using behavioural studies and trials, as solely relying on these correlative approaches can lead to unexpected conclusions (e.g. Tosetto et al., 2021).

Beyond the chromatic component of perception, organisms also vary widely in their ability to visually resolve details from an object or scene; termed ‘visual acuity’ (Caves et al., 2018). Lower levels of visual acuity mean an organism resolves less details of an object or scene being viewed. Acuity therefore has a clear impact on the interpretation of results in studies that are testing behavioural responses to certain stimuli, or the functional implications of certain colour patterns or signals (Caves et al., 2016). Many organisms have acuity much worse than our own, so it is important to consider when assessing how colours and patterns are perceive by other organismal viewers (Caves et al., 2019).

Rather quickly it becomes quite apparent that vision and perception vary widely within the natural world. Thus, it is critically important to know: (1) if your research question involves an explicit viewer and (2) if so, what are their visual capabilities (chromatic, achromatic and acuity) and how do they need to be considered. Previous syntheses in colour research show that most studies come from one of two schools of inquiry: ‘bottom‐up’ and ‘top‐down’ (Kemp et al., 2015). ‘Bottom up’ research questions ‘seek to understand the proximate basis of colour propagation, reception and perception’. These disciplines aim to form a physical and neural understanding of how colour is viewed and processed. Thus they often involve a model study taxon whose vision and perceptive abilities are studied in great detail (Tosetto et al., 2021). ‘Top‐down’ approaches ‘seek to use colour as a trait in tests of ecological and/or evolutionary hypotheses’. These studies often take a broader approach and look at entire groups of organisms simultaneously to understand broad patterns shaping phenotypes through space and time (Cooney et al., 2022). Frequently, ‘top‐down’ questions do not approach their research from the perspective of a specific viewer and therefore stick to more descriptive methods for characterising colours. Thus, it is critically important to identify if your research is a discriminatory/perceptual question (involving a specified viewer) or a spectral/physical question (describing broader patterns pertaining to light and colour). We use this dichotomy in the main methods figure to help identify what type of question certain techniques can be used to answer (Kemp et al., 2015). A large resource table (Table 1, described in more detail below) also lists whether applications are spectral/physical, discriminatory, or perceptual in nature. Sometimes simple approaches and metrics of colouration work fine for the question being asked. It ultimately always hinges on the specific research question being addressed.

**TABLE 1:** Resources for handling, manipulating and analysing images for colour pattern analyses.

Goal	Technique	Description	Function name	Resource	Approach	Reference(s)
Colour standardisation	Linearise reflectance	Linearises reflectance of images based on grey standards and standardises exposure	*‘Generate Multispectral Image’* *‘Model Linearisation Function’*	MICA^a^	S/P/D	Troscianko and Stevens (2015)
	Calibrate colours across images	Calibrate image colours using a commercial colour standard that has been included in the image	*‘colorChecker’*	Patternize^b^	S/P/D	Van Belleghem et al. (2018)
Alter images	Colour segmentation	Identify *n* clusters of colours that most accurately define an image based on either the visual capabilities of a viewer, properties of the colour patches, or parsimony	*‘Naive Bayes Quantisation’* *‘RNL Clustering’* *‘Cluster Particle Analysis’* *‘recolorize’* *‘classify’* *‘getKMeanColors’*	QCPA^a^ Recolorize^b^ PAVO^b^ Colordistance^b^	P/D	Maia et al. (2019), van den Berg et al. (2019), Weller (2021), Weller and Westneat (2019)
	Separating subject from the background	Uses machine learning approaches to identify and crop subjects from backgrounds	sashimi.py (script)	Sashimi^c^	S	Schwartz and Alfaro (2021)
	Simulating acuity	Simulate visual acuity on an image as it would be seen by a viewer. Sharp edges can be reconstructed to simulate acuity more accurately. Requires estimates of the proposed viewer's visual acuity	*‘procimg’* *‘Acuity View’* *‘Gaussian Acuity Control’* *‘RNL Ranked Filter’*	PAVO^b^ QCPA^a^	D	Caves and Johnsen (2018), Maia et al. (2019), van den Berg et al. (2019)
	Simulate false colours	Create display images that simulative perceptive differences between various	*‘Make Presentation Image’*	QCPA^a^	P/D	van den Berg et al. (2019)
	Recover underwater image colours	Algorithm that ‘removes’ the water from images, restoring colour as it would be seen at the surface	seathru.py (script)	Sea‐Thru^c^	S	Akkaynak (2019)
Perceptually compare colours	Receptor‐noise limited (RNL) model	Computes Receptor Noise limited model (Vorobyev & Osorio, 1998) to assess discrimination between colours	*‘vismodel’* *‘RNLmodel’* *‘RNLthresh’* *‘Run QCPA’ ‘Framework’*	PAVO^b^ Colourvision^b^ QCPA^a^	P/D	Gawryszewski (2018), Maia et al. (2019), van den Berg et al. (2019)
	Measure perceptual distance between colours	Measures the perceptual distances between colours within the framework of a visual model	*‘coldist’* *‘Colour/Luminance JND’* *‘Difference Calculator’* *‘deltaS’*	PAVO^b^ MICA^a^ Colourvision^b^	P	Gawryszewski (2018), Maia et al. (2019), Troscianko and Stevens (2015)
Plot/Compare colours graphically	Spectral distributions	Plotting of spectral data typically recorded from spectrometers. This depicts a distribution of reflected light across a wavelength range	*‘procspec’* *‘explorespec’* *‘plotsmooth’*	PAVO^b^	S	Maia et al. (2019)
	RGB, HSV, CIELab Colour spaces	Plot colours based on their coordinates within predefined colour spaces. Compatible with RGB, HSV and CIELab	*‘plotPixels’* *‘plotHist’* *‘colspace’*	Colordistance^b^ PAVO^b^	S/P/D	Maia et al. (2019), Weller and Westneat (2019)
	Receptor‐based colour space	Plot colours based on how they stimulate various photoreceptors within the retina of a viewer. Examples include the Receptor‐Noise limited (RNL) colour space, Chittka colour hexagon and the colour tetrahedron	*‘CTTKmodel’* *‘EMmodel’* *‘RNLmodel’* *‘colspace’*	Colourvision^b^ PAVO^b^	P/D	Chittka (1992), Endler and Mielke (2005), Gawryszewski (2018), Maia et al. (2019)
	Colour maps	An extension of the RNL Model, where all pixel colours from an image can be plotted in a perceptual space	*‘RNL Colour Maps’*	MICA^a^	P/D	Troscianko and Stevens (2015)
Summarise full colourations	Compare pixel colour distribution	Extracts pixel colour data from each image and compares the distribution of colours between images	*‘getImageHist’* *‘getColorDistanceMatrix’*	Colordistance^b^	S/P/D	Weller and Westneat (2019)
	Location‐based colour pattern extraction	Detects the distribution of colours on an organism with respect to its location on the body. Some techniques use landmarks to make comparisons between individuals with different shapes	*‘patLanRGB’* *‘patRegRGB’* *‘rgb.measure’* *‘Marking Matrix’*	Patternize^b^ Colormesh^b^ PAT‐GEOM^a^	S	Chan et al. (2019), Valvo et al. (2021), Van Belleghem et al. (2018)
Compare entire colour patterns graphically	Plotting colour pattern ordinations	Uses ordination techniques (PCA/RDA/MDS) to plot samples in ‘colour pattern’ space. Proximity of points corresponds to similarity in colour pattern	*‘patPCA’* *‘patRDA’* *‘getColorDistanceMatrix’*	Patternize^b^ Colordistance^b^	S	Van Belleghem et al. (2018), Weller and Westneat (2019)
Quantify colour space occupancy	Measure colour space volume	Measure volumes occupied by colours/patterns in a various spaces. Some applications can measure concave hyper volumes using α‐shapes	*‘vol’* *‘tcsvol’* *‘voloverlap’*	PAVO^b^	S/P/D	Gruson (2020), Maia et al. (2019)
Measure visual and geometric aspects of pattern elements	Characterise pattern patch aspects	Resources that aim to describe primarily geometric aspects of colouration. These include functions that range from measuring patch size, complexity and direction to the area of certain colours on a body and where they are distributed across multiple individuals and species	*‘Elliptical Fourier Shape’* *‘Analysis’* *‘Directionality of Shape’* *‘Centroid Size’* *‘Contrast CoV’* *‘patArea’* *‘plotHeat’* *‘Cluster Particle Analysis’*	PAT‐GEOM^a^ Patternize^b^ QCPA^a^	S	Chan et al. (2019), Van Belleghem et al. (2018), van den Berg et al. (2019)
	Colour Adjacency Analysis	Analyse the frequency of colour transitions within a region of interest	*‘Run QCPA Framework’* *‘adjacent’*	QCPA^a^ PAVO^b^	S/D	Endler (2012), Maia et al. (2019), van den Berg et al. (2019)
	Boundary Strength Analysis	Analyses the visual strength of changes at boundaries between various elements of a colour pattern	*‘Run QCPA Framework’* *‘adjacent’* *‘recolorize_adjacency’*	QCPA^a^ PAVO^b^ Recolorize^b^	D	Endler et al. (2018), Maia et al. (2019), van den Berg et al. (2019), Weller (2021)
	Local Edge Intensity Analysis &Disruption	Measures the intensity of chromatic and achromatic changes across image elements	*‘Run QCPA Framework’* *‘Gabrat Disruption’*	QCPA^a^	D	van den Berg et al. (2019)

*Note*: In the column ‘Approach’, S – Spectral/Physical, P – Perceptual distance and D – Discriminatory techniques following Kemp et al., (2015).

^a^ Resource in ImageJ.

^b^ Resource in R.

^c^ Resource in Python.

A strong understanding of the concepts summarised above will better inform the experimental design, how data is collected and analysed, and most importantly, its interpretation (Endler & Mappes, 2017). Herein, most of the material presented and its proposed uses will be from a ‘top‐down’ perspective as these questions are more likely in broader ecological and evolutionary studies. However, it is up to the researcher to perform their own due diligence and ensure that they have a firm grasp of the relevant theory behind their research question before implementing, analysing and interpreting their findings.

### 2.2 An overview of the resources available

**FIGURE 1:**
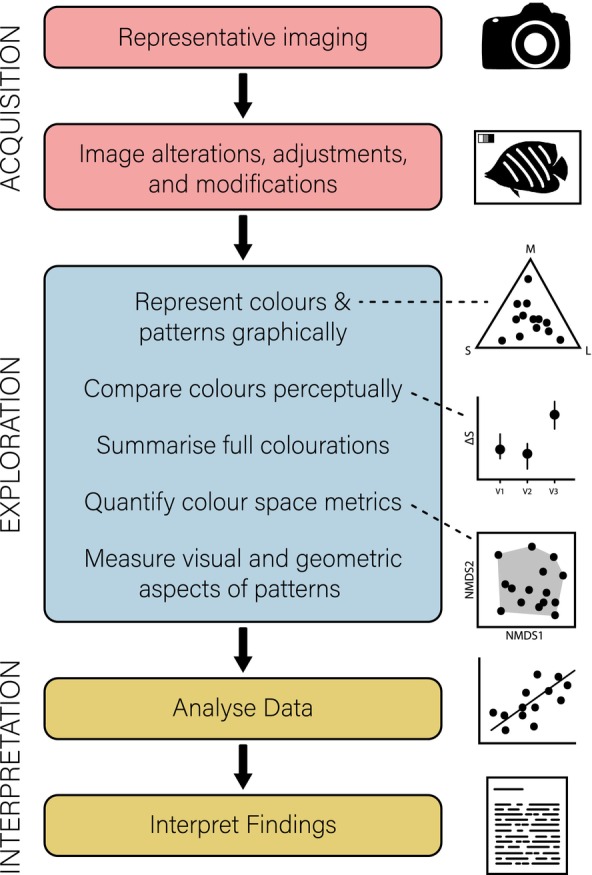
The typical workflow in the study of biological colourations from digital images. The steps in red represent those prior to analyses. Blue are data exploration and interpretation techniques. Yellow are the final steps of analysing the data and formulating conclusions.

Following is an overview of the current resources available for image processing and analysis in colour pattern studies with a focus on those available in R and ImageJ. The resources have been organised in a manner that would mirror a typical workflow when analysing colours and patterns from digital imagery (Figure 1). A current subset of some available methods, including their function description, name, reference for further reading and platforms on which they are available is listed in Table 1 as well as a visual overview in Figure 4. The presentation of resources in the table has also been organised to mirror a typical workflow in image analysis. Last, throughout the text, the specific functions, feature, or tool used to perform each technique is denoted with *italic* text.

### 2.3 Image processing prior to analysis

Images form the foundations upon which most of the analyses and techniques covered herein are based. Therefore, it is crucial that images have been taken in a standardised and representative manner. Ideally, images are captured in a *raw* format; an image format where the camera makes minimal changes to the image preserving as much of the original scene's light information as possible. These formats offer the greatest flexibility and most accurately represent the true appearance of an object or scene. More common file types, like *.jpeg* are compressed meaning file information is deliberately discarded to reduce file size. Additionally, irreversible changes are often made to these images by the camera's processor which alter the photograph in ways that are thought to make it look more pleasing to the viewer. This typically includes boosting the saturation and vibrancy or altering the contrast of the colours within an image. Clearly, this poses a problem if the object of a study is to compare images objectively. For comprehensive guides to digital imaging for the study of biological colouration and their limitations, please see Stevens et al. (2007) and White et al. (2015).

**FIGURE 2:**
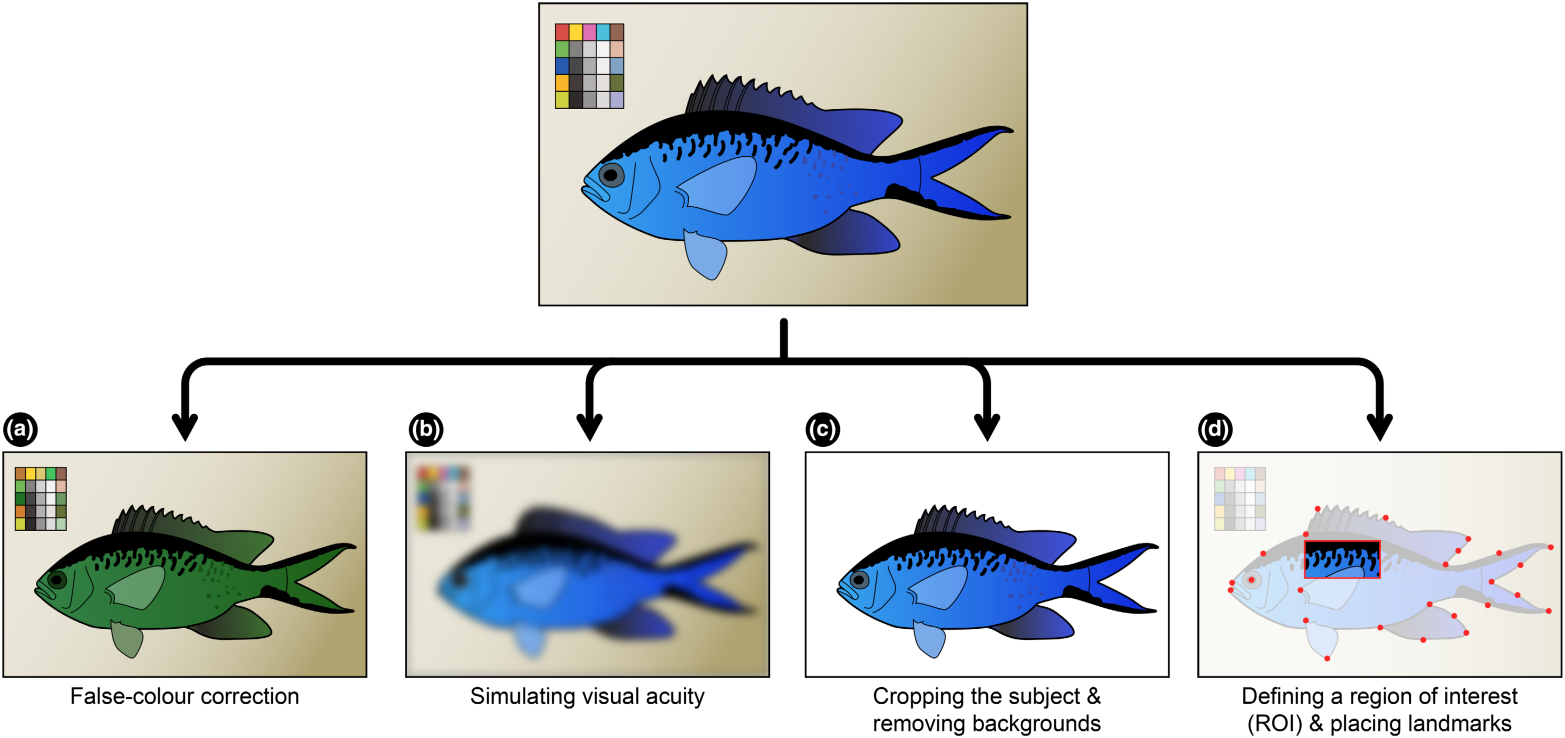
Some of the alterations that can be made to images prior to analysis. (a) changing the colours within an image to create a false‐colour photograph to highlight discriminability, (b) adjusting an image to reflect a given viewers visual acuity, (c) cropping a subject to remove its background and (d) defining a region of interest (ROI) for the analysis or placing landmarks. These techniques are not mutually exclusive and often multiple will be combined depending on the research question.

Often the first step after imaging for most colour‐based research questions will involve processing and manipulating images in various ways to prepare them for analyses (Figure 2). Colour and grey standards are small items included in images that contain specific colours of known reflectance and hue. If standards have been included in the images, then the image's colours can be adjusted to ensure the lighting has been standardised/normalised between all photos. This is particularly important when photographs were taken outdoors where cloud cover and time of day can greatly impact the available light spectrum (Bergman & Beehner, 2008; Stevens et al., 2007). Images containing the Calibrite (formerly X‐Rite) ColorChecker Passport or the Image Science Associates ColorGauge can be adjusted using the function (‘*colorChecker*’) within the patternize R package (Van Belleghem et al., 2018). Generally, *.jpeg* and other non‐*raw* file types are nonlinear in nature, meaning the brightness of some pixels are increased or decreased more relative to others. To linearise these images (that is, to make the brightness more accurately reflect the number of photons hitting the cameras sensor) the images must include a grey standard. The linearisation can be done using the Multispectral Image Calibration and Analysis (MICA) toolbox (Troscianko & Stevens, 2015) using ‘*Model Linearisation Function*’. Calibrating images in the ultraviolet or infrared regions of the spectrum will require special standards that have UV/IR reflective properties as most commercial options only reflect visible light.

Once images are colour‐accurate and representative, further changes can be made to mimic how certain organisms may perceive the scene photographed within each image (Troscianko & Stevens, 2015). Every organism possesses its own unique assemblage of photoreceptor cells giving it the ability to perceive light and certain colours (Kelber et al., 2003; Osorio & Vorobyev, 2008). The MICA toolbox and the Quantitative Colour Pattern Analysis framework (QCPA) provide a suite of resources that analyse colours and patterns from an explicitly visual perspective (Troscianko & Stevens, 2015; van den Berg et al., 2019). To use this approach, knowledge of the spectral sensitivities of the taxon of interest are required (discussed in more detail below). The ‘False‐Colour’ images (Figure 2a) which can be made in the MICA Toolbox (‘*Make Presentation Image*’) attempt to give an impression of the relative discriminability of a scene to a specific viewer, but not imitate what an organisms would actually see (van den Berg et al., 2019; Verhoeven et al., 2018). Although these images are generally for demonstration purposes only, they can identify some unique aspects to colouration that humans would not natively perceive. For example, showcasing a range of unique patterns found on flowers that possibly act as signals to attract pollinators (Lunau et al., 2021).

If your research question is discriminatory in nature (for example, how well can a predator detect a prey item from a certain distance), then visual acuity may need to be incorporated into the analysis (Figure 2b). Caves and Johnsen (2018) were first to develop an algorithm and associated R package (AcuityView) dedicated to simulating acuity. The user specifies: (1) the visual acuity of the viewer (in cycles per degree or minimum resolvable angle) and, (2) the distance between the subject and the viewer. A fast Fourier transformation is then performed to remove static spatial details that a viewer would not likely resolve from a scene. The original AcuityView algorithm has been updated and can now be implemented in both the QCPA framework (‘*Acuity View’*) and PAVO (‘*procimg*’) (Maia et al., 2019; van den Berg et al., 2019). Furthermore, the QCPA framework can also simulate acuity using a different approach (‘*Gaussian Acuity Control*’) that works on non‐rectangular regions of interest (unlike AcuityView) offering greater flexibility.

Images may need to have the subject cropped from the background or a region of interest denoted to facilitate further analyses (Figure 2c,d). Cropping the subject is most easily done in Adobe Photoshop using the ‘*Quick Selection*’ tool. For those with more programming experience, various machine learning pipelines can be used (typically in Python) to automatically detect and segment the subject from the background (Schwartz & Alfaro, 2021). Alternatively, your research question may only be concerned with a specific region within an image. Depending on the downstream analyses being performed, you may either need to manually draw the outline for the region of interest (ROI) or supply a file (typically a text file) containing the coordinates that denote the ROI. (Figure 2d). In these instances, downstream analyses are only performed on the area within the ROI. Lastly, your research question may require the placement of landmarks to align multiple images (Van Belleghem et al., 2018). Landmarks can easily be placed in ImageJ using the ‘*point*’ or ‘*Multi‐point*’ tool (Figure 2d). After placing landmarks points, the *x* and *y* coordinates of all points can be exported and saved as a text file or spreadsheet.

### 2.4 Representing colours graphically

**FIGURE 3:**
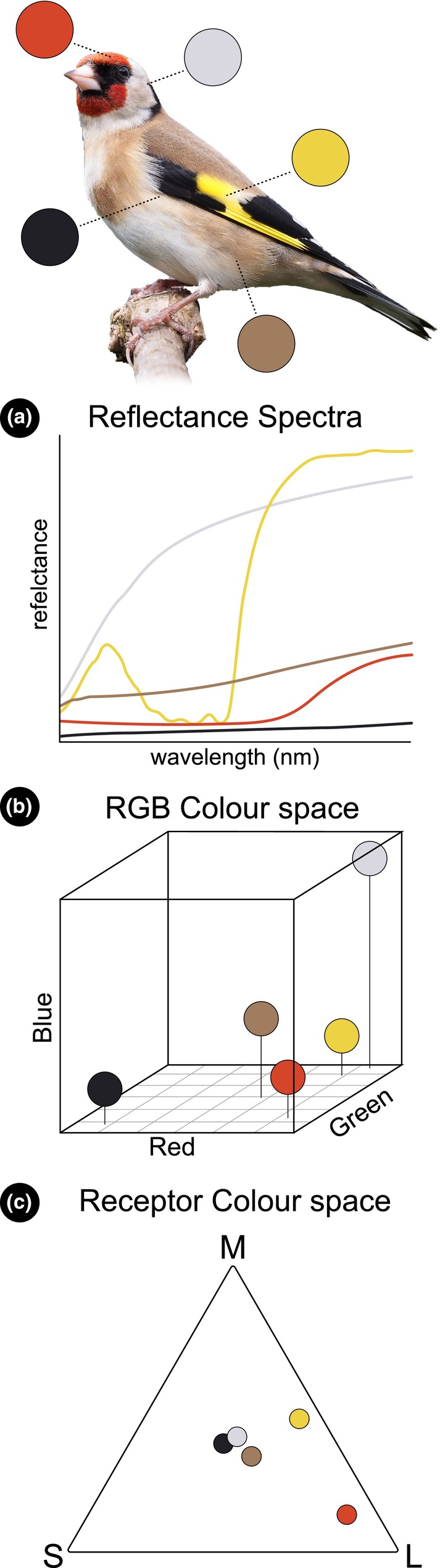
Three alternative methods to graphically represent colours. The top is an image of the European Goldfinch (*Carduelis carduelis*). Five colours have been sampled across its body. (a) Colours represented as a distribution of the relative amount of reflected light at each wavelength. Spectral reflectance data are reproduced from Stavenga and Wilts (2014). (b) Colours represented in the RGB colour space. (c) Colours represented in hypothetical receptor space by how strongly they stimulate three photoreceptor types that are sensitive to short (S), medium (M) and long (L) wavelengths. Photo: Francis C. Franklin. CC BY‐SA 3.0.

Representing colours graphically allows for additional unique interpretations and analyses to be made with colour data. Which plotting technique is most appropriate depends entirely on the data and approaches used. The most fundamental method for plotting the physical properties of a colour is by displaying it as a reflectance spectrum (Figure 3a). This is the relative amount of light at specific wavelengths that have been reflected off of a surface or object; typically measured using a spectrometer (Endler, 1990). This method is particularly useful for initially comparing specific colours to known spectral sensitivities of certain photoreceptors within a viewer (Johnsen, 2016; Kelber et al., 2003). Although this is not a method in which patterns are assessed nor is it collected using digital imagery (although new techniques are emerging that can reconstruct reflectance spectra from digital images; Deng et al., 2021; Zhao & Berns, 2007), it is worth mentioning due to its specific applicability and longstanding use in the field (Endler, 1990) The PAVO (Perceptual Analysis, Visualisation and Organisation of spectral colour data) R package provides easy‐to‐use resources for plotting and visualising spectral data (‘*explorespcec*’) (Maia et al., 2013, 2019). Visualising colours using this approach can also identify possible latent properties about the object/organism being studied. For example, how two seemingly identical colours can be created from fundamentally different spectral distributions (called ‘metamerism’; Endler, 1990).

An extension beyond plotting spectral distributions are colour spaces. Colour spaces are graphical techniques used to arrange colours spatially based on a set of criteria within a *n*‐dimensional coordinate system (Renoult et al., 2017). The axes of the coordinate systems differ depending on the rules used to construct the space; whether it be based on how humans perceive or categorise colours (RGB and CIELAB colour spaces) (Weller & Westneat, 2019) to how light stimulates certain photoreceptors within the eye (Chittka, 1992; Endler & Mielke, 2005). From a spectral/physical perspective, the RGB (red, green, blue, Figure 3b) colour space is common in computer graphics which contains three, perpendicular axes (*x*, *y*, *z*) that loosely imitate the three peak spectral sensitivities of photoreceptors in humans (blue – short wavelengths, green – medium wavelengths and red – long wavelengths). While convenient to work with in digital settings, distances within this colour space are not representative of perceptual distances, that is, how different we as humans would perceive two or more colours. To overcome this limitation, the CIELab colour space was intentionally designed so that Euclidean distances between colours closely approximate their perceptual difference in life to humans. The CIELab colour space uses a lightness axis (L), differences along a red–green axis (a) and differences along a blue – yellow axis (b). The R packages colordistance (‘*plotPixels*’) and PAVO (‘*colspace*’) can plot colours within an image or ROI within these colour spaces.

An alternative graphing technique is using *n*‐dimensional spaces whose axes correspond to how certain photoreceptors are stimulated given the capabilities of a specified viewer (Renoult et al., 2017). These receptor‐based colour spaces have the advantage of displaying colours in space by how they are theoretically perceived by a viewer within a psychophysical framework thus adding an additional layer of ecological or behavioural understanding (Troscianko et al., 2016). These spaces are flexible in that the number of axes can be increased or decreased depending on the number of photoreceptor types present in the viewer. One of the most notable and well established visual models is the Receptor Noise Limited model (RNL) (Vorobyev et al., 2001; Vorobyev & Osorio, 1998). This model estimates receptor spectral sensitivity while simultaneously accounting for inherent noise (caused by molecular ‘misfires’, Barlow et al., 1993) within the receptors. Like any model, it has a series of assumptions that need to be made and met which can be found in detail in the original description (for example, that colour is neurally coded using opponent mechanisms). Results from this model can then be plotted in a *n*‐dimensional colour space which can accommodate varying numbers of receptor sensitivities (to date, modelling up to four) making it flexible for many study taxa (Hempel De Ibarra et al., 2001). Importantly, distances between specific stimuli within these colour spaces (i.e. their Euclidean distance termed ΔS) aim to more accurately reflect perceptual distances inherent to the viewers. However, whether these colours are actually perceived differently requires experimental validation. Additional receptor colour spaces that are more generalised or specialised in nature have been created, such as the Tetrahedral Colour Space (Endler & Mielke, 2005) and the Colour Hexagon (Chittka, 1992). These spaces can be implemented in numerous platforms, including the colourvision R package (‘*colspace*’) and the PAVO R package (‘*CTTKmodel*’, ‘*EMmodel*’, ‘*RNLmodel*’, ‘*GENmodel*’).

### 2.5 Comparing the colours and patterns within and between images

**FIGURE 4:**
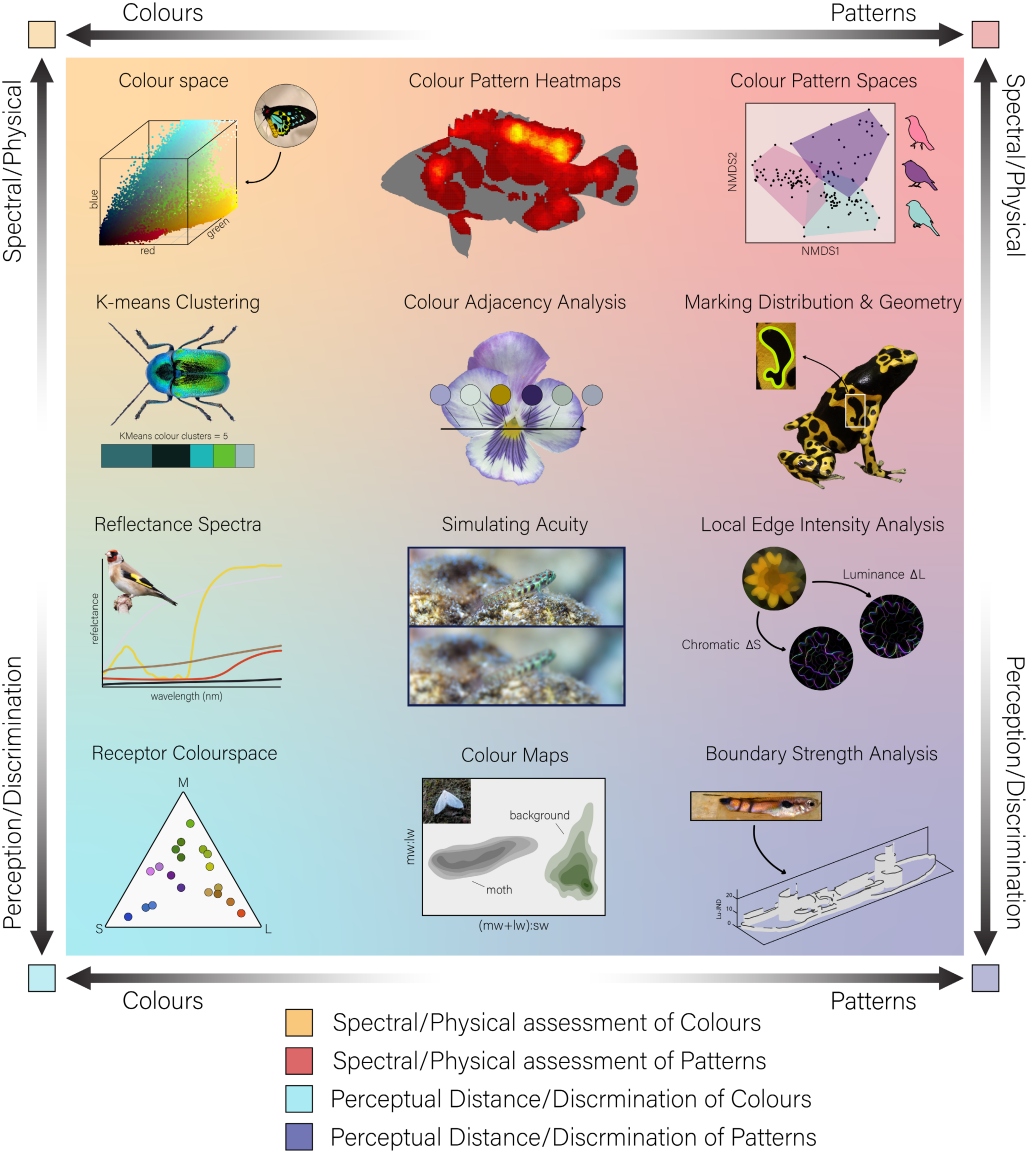
What technique should you use? A sample of analyses are shown which are arranged along the two axes depending on: *y* axis – how one approaches analysing colouration, i.e. spectral/physical or perceptive/discriminatory and the *x* axis –whether one focuses on colours or patterns. Photos credits: H. Krisp, U. Schmidt, F. Franklin, V. Huertas and K. Schulz; CC BY‐SA 2.0. The figures for Local Edge Intensity Analysis, Boundary Strength Analysis and Colour Maps have been adapted from the original publications.

Some research questions may aim to analyse many or all colours and patterns within an image or between multiple images (examples listed in Figure 4). Importantly, these techniques move beyond the traditional approaches of ‘patch‐measuring’ and seek to analyse the complete appearance of an organism or scene in its entirety. The MICA toolbox provides an extension of the Receptor Noise Limited model, called ‘Colour Maps’, which can plot millions of pixel's colours from an image into a single psychophysically calibrated colour space (‘*RNL Colour Maps*’). The boundaries around colour points (in a similar fashion to error bars) can be adjusted to represent different amounts of discriminatory distance (Just Noticeable Difference). For example, this space could be used to initially explore how a Honeybee (*Apis mellifera*) might distinguish the colours of a flower as compared to the background vegetation. This information may then be used to formulate hypotheses that can be experimentally tested and ground‐truthed using behavioural trials. In the case of the honeybee, these methods have shown the visual salience of a flower from its background is indeed indicative of its detection success (Spaethe et al., 2001).

Alternatively, you may want to measure and compare the distribution and amount of all colours found within one image to another (or many). The R package colordistance can quantitatively evaluate the similarity between the distribution of colours among *n* images (Weller & Westneat, 2019). Colordistance characterises colouration by plotting a sample of pixels from an image within a predetermined colour space (e.g. RGB, CIElab; non‐human perceptual spaces are not supported). The axes within this space are then divided into equal area subsections as specified by the user. The number of pixels that occur within each subsection is then counted and used to create a distribution that can be compared to every other image analysed using multivariate approaches. The intuitive nature of this approach allows for the full colouration of any number of organisms to be compared regardless of morphological or size differences. Furthermore, the user can specify: (1) colour space to be used, (2) how fine the resolution of colours are (i.e. the number of ‘subsections’ to divide the colour space into) and (3) the method to compare the distribution of colours (e.g. earth movers distance, *χ* ^2^ distance, etc.). However, the use of strictly human‐related colour spaces limits its applicability to more human‐centric questions.

If the exact location of where each colour occurs is important (i.e. pattern), colormesh offers utilities to efficiently measure colours across the body of the study subject (Valvo et al., 2021). First, images are unwarped using landmarks to match a consensus shape which allows colours to be compared at the same location across individuals whose morphology and shape may vary, or between images in which the subject's orientation differs. Delaunay triangulation creates a mesh across the body which (which can be changed depending on how fine or coarse the user would like to sample) is then used to select relatively even and representative sampling locations across the organisms being assessed. The RGB values of the pixel at the centre point of each triangle are then recorded (*‘rgb.measure’*) and can be converted into a data matrix (‘*make.colormesh.dataset*’) for further exploration and analyses. Colormesh does offer functions to implement image linearisation as well as calibration to known colour standards (‘*rgb.calibrate*’). However, this is the only colour space currently supported so thoughtful consideration is needed when deciding to use this approach.

The R package patternize also offers utilities to analyse colour patterns with respect to morphological location (Van Belleghem et al., 2018). patternize can use landmarks, in a similar fashion to colormesh, to align images but can also perform image registration where images are automatically aligned. The RGB triplet (this is the only colour space supported) value of the colour of interest, along with a tolerance parameter (that allows for a range of RGB values above and below the target value) are specified and detected in each image. While patternize was developed to work with only one colour, the source code can be modified to permit its use with multiple colours (e.g. Hemingson et al., 2019). The output of this analysis can be visualised and compared using multiple different techniques. These range from plotting the heat maps of specific colours across the bodies of multiple organisms (‘*plotHeat*’) to comparing the colour matrices using multivariate approaches (‘*patLanRGB*’) and visualising them using ordination techniques (‘*patPCA*’).

The plotting of both individual colours (techniques described in the previous section) as well as entire colours and patterns in reduced dimensional spaces allows for further metrics to be measured that summarise and describe and organism's colouration. One of the most notable is their *n*‐dimensional volume. This is frequently the convex or concave hull volume (among others, Mouillot et al., 2013), which are alternate methods for measuring the volume occupied by a set of points. These techniques have a long standing use in the literature and can function as simple indices of colour diversity (Gruson, 2020). A myriad of other metrics exists that aim to summarise and characterise multivariate data; mostly developed by community ecologists aiming to describe community composition (Legendre et al., 2005; Mouillot et al., 2013). These metrics can easily be adapted to work for multivariate colour data and offer an exciting new field of inquiry for this research. Recently, these metrics have been used to measure the diversity of colours found on individuals to analyse global trends in colourfulness (Cooney et al., 2022), as well as comparing the collective colourations of all individuals found in different habitats (Hemingson et al., 2022).

### 2.6 Measuring the visual and geometric aspects of pattern

There are many applications that analyse various geometric or spatial aspects of colouration within an image. These techniques explicitly incorporate the location of colours into the analyses and, depending on the technique, can include the visual capabilities of a specified viewer. These approaches are not restricted to only analysing colour patterns at the scale of the individual (i.e. the colouration on a frog), but can also assess how colours change between elements within an image (e.g. the foreground vs background). For example, some of these techniques could be used to assess the theoretical perceptual contrast between a subject when compared to its environment e.g. a red flower to a green, foliage dominated background. In this example, the techniques are not assessing pattern per se, but rather assessing the difference between colours with respect to their location within an image.

Boundary Strength Analysis (Endler et al., 2018), Local Edge Intensity Analysis (‘*Run QCPA Framework*’) (van den Berg et al., 2019) and Gabor Ratios (‘*Gabrat Disruption*’) are all useful techniques to identify the intensity of colour changes between elements within an image. These approaches work by modelling both the chromatic and luminance differences (ΔS and L) between different colours and elements. Each technique will have output tables that summarise the differences calculated within the image. The original images can also be visualised using plots that overlay the visual intensity of changes between colours/elements. The strength of these changes will be entirely context dependent on the visual capabilities of the viewer being modelled.

If the research question is more focused on characterising the complexity of a colour pattern at the scale of the individual (i.e. the colour pattern of an orchid flower – see van den Berg et al., 2019), Colour Adjacency Analysis (‘*Run QCPA Framework*’ in QCPA, ‘*adjacent*’ in PAVO) offers simplified summary metrics. This analysis runs multiple transects across the region of interest in both the *x* and *y* dimensions. The colour is recorded at set intervals along each transect. These transects are then summarised and used to create a transition matrix that contains how often colour changes along all transects (Endler, 2012). This is a useful technique to simply characterise the complexity of a colour pattern. The output is a single value than can then be used in further downstream analyses. However, if the colours between two images are different but the pattern is the exactly the same, the Colour Adjacency metric for both images will be identical (see van den Berg et al. (2019) for details). Thus, consideration is needed when using and interpreting this metric.

PAT‐GEOM is an ImageJ plugin that provides multiple tools to measure aspects of pattern geometry (Chan et al., 2019). These range from assessing the shape complexity of individual markings (‘*Elliptical Shape Fourier Analysis*’), marking size, patch directionality (‘*Directionality of Distribution*’), randomness and distribution (‘*Marking Matrix*’). These techniques seek to measure aspects at the marking or individual level. These tools have been used to demonstrate that marking size on three different populations of furrowed crabs (*Xantho hydrophilus*) closely resembled the background of their local environment (Chan et al., 2019). The QCPA framework also offers resources for measuring patch aspects, like the size, shape, distribution and angle of particles within a patch (‘*Cluster Particle Analysis*’).

## 3 CONCLUSIONS AND FUTURE PERSPECTIVES

The strength of these various approaches arises at the intersection of their use – combining aspects to ask higher‐order questions that begin to bridge the gap between ‘bottom‐up’ and ‘top‐down’ approaches. These questions would allow for the consideration of the perceptive and resolving capabilities of individual organisms (‘bottom‐up’) while also comparing multiple individuals or species within an evolutionary or ecological context (‘top‐down’). Fruitful lines of future research range from simulating what entire communities of organisms appear like to specific taxa (like a predator) to identifying macroecological patterns of colouration and how it relates to certain behaviours, like courtship and sexual selection whilst accommodating visual properties of the viewer (Endler & Mappes, 2017).

Many of the applications discussed herein also have use outside of their conventional design. For example, recent research used patternize to construct ‘damage heatmaps’ that display where different predatory reef fish injure their prey (Muruga et al., 2022). Damaged prey fishes were dissected out of the predators shortly after ingestion and were photographed. The injuries on every individual were manually painted onto the images in Photoshop with solid colours. These colours were then detected for and mapped using patternize to show where damage most likely occurred for each different predator type showing that predators with different tooth morphologies generally capture and process prey differently.

New hardware is revolutionising the field. Digital cameras of increasingly higher quality are becoming cheaper and open‐source designs for spectrometers and other equipment are now available, drastically reducing the initial startup costs of working in this field (Caves et al., 2020; Troscianko, 2022). Hyperspectral cameras capture the entire spectral distribution inherent to each pixel within an image, as opposed to the relative amount of light within specific wavelength bands (e.g. RGB). Hyperspectral images contain immense amounts of raw data compared to those taken by traditional cameras and can be used to further ask interesting questions about colouration in the natural world (Garcia et al., 2015). For example, tuning a hyper spectral camera to mimic the spectral sensitivity of a specific taxon to take images that closely resemble what that taxon would likely see. While the use of these cameras is still in their infancy in the life sciences due to their high cost (Zimmermann et al., 2018), there will likely be a transition to these devices over traditional cameras as they become more affordable.

Machine learning approaches are also rapidly changing the field (Fennell et al., 2021). Mentioned previously, various pipelines offer the ability to accurately detect and segment focal taxa form their backgrounds using convolutional neural networks (Schwartz & Alfaro, 2021). Different datasets can be used to train the model allowing for widespread use on many study groups. Machine learning approaches can also be used to help inform future research questions. The CamoEvo toolbox is an open access resource that is used to study the evolution of camouflage (Hancock & Troscianko, 2022). Users play an interactive game in which the subject (a simulated sphere) should be selected as fast as possible from a suite of background images. This data is then fed into an algorithm that alters the colouration to maximise the time taken to be selected – mimicking natural selection. Resources like this can be used to hone in on the selective pressure shaping camouflage patterns and then be ground‐truthed using field experiments (e.g. Kjernsmo et al., 2020). These approaches show much promise for the field of colour science.

The multitude of recent advances has made the field of organismal colouration exciting to study. By combining old and new techniques from different backgrounds, we are now capable of asking detailed questions about the appearance of organisms and how they are perceived. The goal of this review is to provide a starting point to help researchers navigate the methodologically dense field of biological colouration. We must be explicit, however, and reiterate that it is imperative to have a knowledge background relevant to one's research focus. Without a solid foundation, it is easy to make conclusions that are misleading and are not grounded in theory. Research in this field can be a unique blend of physics, biology, psychology, behaviour and ecology. Thus, the necessary background knowledge needed will be specific to your research question.

Future research is likely to yield new ways of thinking about colouration (Garcia et al., 2020). In just the last 5 years, there have been numerous developments and modifications made to existing techniques to answer interesting new questions. By combining new ways to assess colouration and further refining visual modelling, we are gaining an increasingly comprehensive understanding of how colouration functions in the natural world.

## AUTHOR CONTRIBUTIONS

**Christopher R. Hemingson:** Conceptualization (lead); data curation (lead); formal analysis (lead); funding acquisition (supporting); investigation (lead); methodology (lead); project administration (equal); resources (lead); software (lead); validation (lead); visualization (lead); writing – original draft (lead); writing – review and editing (lead). **Peter F. Cowman:** Methodology (supporting); project administration (supporting); supervision (supporting); writing – original draft (supporting); writing – review and editing (supporting). **David R. Bellwood:** Funding acquisition (lead); project administration (supporting); supervision (lead); validation (supporting); writing – original draft (equal); writing – review and editing (equal).

## CONFLICT OF INTEREST STATEMENT

The authors declare that there are no conflicts of interest.

## Data Availability

Our publication is a review and has not associated data.
